# Oral Administration of *p*-Hydroxycinnamic Acid Attenuates Atopic Dermatitis by Downregulating Th1 and Th2 Cytokine Production and Keratinocyte Activation

**DOI:** 10.1371/journal.pone.0150952

**Published:** 2016-03-09

**Authors:** Hyun-Su Lee, Eun-Ju Choi, Kyung-Sik Lee, Hye-Ran Kim, Bo-Ra Na, Min-Sung Kwon, Gil-Saeng Jeong, Hyun Gyu Choi, Eun Young Choi, Chang-Duk Jun

**Affiliations:** 1 School of Life Sciences, Immune Synapse Research Center and Cell Dynamics Research Center, Gwangju Institute of Science and Technology, Gwangju, Republic of Korea; 2 Division of Sport Science, College of Natural Sciences, Konkuk University, Chungju, Republic of Korea; 3 College of Pharmacy, Keimyung University, Daegu, Republic of Korea; 4 College of Pharmacy, Yeungnam University, Gyeongsan, Republic of Korea; 5 Department of Biomedical Sciences, University of Ulsan College of Medicine, Seoul, Republic of Korea; Hanyang University, REPUBLIC OF KOREA

## Abstract

Atopic dermatitis (AD) is a complex disease that is caused by various factors, including environmental change, genetic defects, and immune imbalance. We previously showed that *p*-hydroxycinnamic acid (HCA) isolated from the roots of *Curcuma longa* inhibits T-cell activation without inducing cell death. Here, we demonstrated that oral administration of HCA in a mouse model of ear AD attenuates the following local and systemic AD manifestations: ear thickening, immune-cell infiltration, production of AD-promoting immunoregulatory cytokines in ear tissues, increased spleen and draining lymph node size and weight, increased pro-inflammatory cytokine production by draining lymph nodes, and elevated serum immunoglobulin production. HCA treatment of CD4^+^ T cells *in vitro* suppressed their proliferation and differentiation into Th1 or Th2 and their Th1 and Th2 cytokine production. HCA treatment of keratinocytes lowered their production of the pro-inflammatory cytokines that drive either Th1 or Th2 responses in AD. Thus, HCA may be of therapeutic potential for AD as it acts by suppressing keratinocyte activation and downregulating T-cell differentiation and cytokine production.

## Introduction

Atopic dermatitis (AD) is a multifactorial, complex, and incurable skin disease. Its causes include immune system collapse, genetic defects, and environmental factors [1]. Several mouse models of AD have been developed. Some involve genetic engineering, while others are based on sensitization with allergen [2]. The most commonly used allergenic combination is 2, 4-dinitrochlorobenzene (DNCB) followed by *Dermatophagoides farinae* (mite) extract. Mite extract elicits AD symptoms in 30–50% of patients with AD [3]; when the ears of mice are repeatedly exposed to DNCB and mite extract, they develop many of the characteristic clinical and immunological features of AD. This DNCB/mite extract-induced AD model was employed in the present study.

Once an allergen invades the skin, it is captured by Langerhans cells (LC) activated by keratinocytes which produce a set of proinflammatory cytokines in the inflamed tissue. Captured allergen is processed and presented on the cell surface in the context of major histocompatibility complex class II molecules. Naïve T cells that recognize the antigen on the LCs in the cytokine milieu of T cell-derived interleukin (IL)-4 and IL-10 and activated keratinocyte-derived thymic stromal lymphopoietin (TSLP) differentiate from Th0 cells into Th2 cells. These cells then produce abundant amounts of Th2 cytokines, including IL-4, IL-5, IL-13, and IL-31. This strong Th2-cell response induces acute AD within 24 h of allergen invasion [3,4]. During this process, the LCs also produce chemoattractants such as monocyte chemotactic protein 1 that induce recruitment of monocytes into the inflamed tissue [5]. As the disease progresses to the chronic phase, the recruited monocytes release IL-1, IL-6, tumor necrosis factor (TNF)α, IL-12, and IL-18, which promote a switch from the initial Th2 response to a Th1 type-immune response [6]. Keratinocytes-derived proinflamamtory cytokines activate LCs, i.e., dendritic cells and amplify inflammation in the tissues, thus initiating and maintaining AD [7]. Since Th1/Th2 differentiation and their functions as well as keratinocyte activation are critical to AD development, an ideal therapeutic approach to AD may be a treatment with inhibitors that modulate T cell and keratinocyte activation and thus ameliorate AD symptoms.

Since many people suffer from AD skin diseases globally, there has been considerable research into safe, economically viable, and readily manufactured therapies for AD. In the present study, we assessed the anti-AD properties of *p*-hydroxycinnamic acid (HCA), which can be isolated from *Curcuma longa*. This compound is structurally similar to other phenolic compounds, such as gallic acid [8], coniferyl aldehyde [9], caffeic acid [10], and ferulic acid [11], all of which have been reported to have medicinal properties, including anti-inflammatory [12], anti-microbial [13], hypolipidemic [14], anti-mutagenic [15], and anti-carcinogenic functions [15,16]. In particular, gallic acid, which is a particularly well-studied phenolic compound, has been shown to possess anti-allergic and anti-inflammatory effects: specifically, it inhibits the histamine release and pro-inflammatory cytokine production by mast cells [8]. We recently reported that HCA inhibits T-cell activation by modulating the protein kinase C (PKC) theta (PKCθ) pathway [16]. Here, we investigate the therapeutic potential of HCA in T cell-mediated AD.

## Materials and Methods

### Mice

Eight-week-old female BALB/c mice and C57BL/6 mice were purchased from Samtako and housed in specific pathogen-free conditions. All experiments were approved by the Animal Care and Use Committee of the School of Life Sciences, Gwangju Institute of Science and Technology. The approval number is GIST2014-31.

### Preparation of HCA from the roots of *C*. *longa*

HCA was isolated from the roots of *C*. *longa* by Dr. Seung-Ho Lee, Yeungnam University [17]. Briefly, the air-dried rhizomes (6 kg) were extracted with methanol (10 L) at room temperature for 5 days. The extract (1.2 kg) was then suspended in water and partitioned three times with equal volumes of ethyl acetate. The ethyl acetate extract (70 g) was fractionated by silica gel column chromatography elution with a gradient system of CH_2_Cl_2_-ethyl acetate (from 10:0 to 1:1) to yield seven fractions entitled Frs. 1–7. Fr. 3 (4.3 g), which was particularly enriched, was purified by recrystallization from cold methanol. This resulted in a compound [482.8 mg, 0.69% (w/w)] that was identified to be HCA by using spectroscopic and mass spectrometric analyses and by comparing the results to those in the literature [18].

### Reagents and cell culture

DNCB, mite extract, phorbol 12-myristate 13-acetate (PMA), A23187, and carboxyfluorescein succinimidyl ester (CFSE) were purchased from Sigma (St. Louis, MO). Mouse IgG2a enzyme-linked immunosorbent assay (ELISA) kits, anti-mouse CD4 antibody conjugated with fluorescein isothiocyanate (FITC), anti-mouse interferon (IFN)γ antibody conjugated with PerCP cy5.5, and anti-mouse IL-4 antibody conjugated with phycoerythrin were obtained from eBioscience (San Diego, CA). Rabbit anti-mouse keratin5 antibody was purchased from Abcam (Cambridge, MA) and rabbit anti-mouse p65 antibody was from Cell signaling technology (Beverly, MA). Mouse IgE ELISA kits, purified anti-mouse IFNγ, anti-mouse IL-4, and anti-mouse IL-12 antibodies were purchased from BD Bioscience (San Jose, CA). Anti-mouse CD28 antibody, mouse IFNγ ELISA kits, mouse IL-4 ELISA kits, and recombinant human IFNγ and TNFα were purchased from R&D (Minneapolis, MN). Recombinant mouse IL-4 and IL-12 were obtained from Peprotech (Hamburg, Germany). 145-2C11 (mouse anti-CD3; CRL-1975) hybridoma cell line and HaCaT (human keratinocyte) were obtained from the ATCC (Manassas, VA).

### Induction of atopic dermatitis in the mouse ear

**Fig 1:**
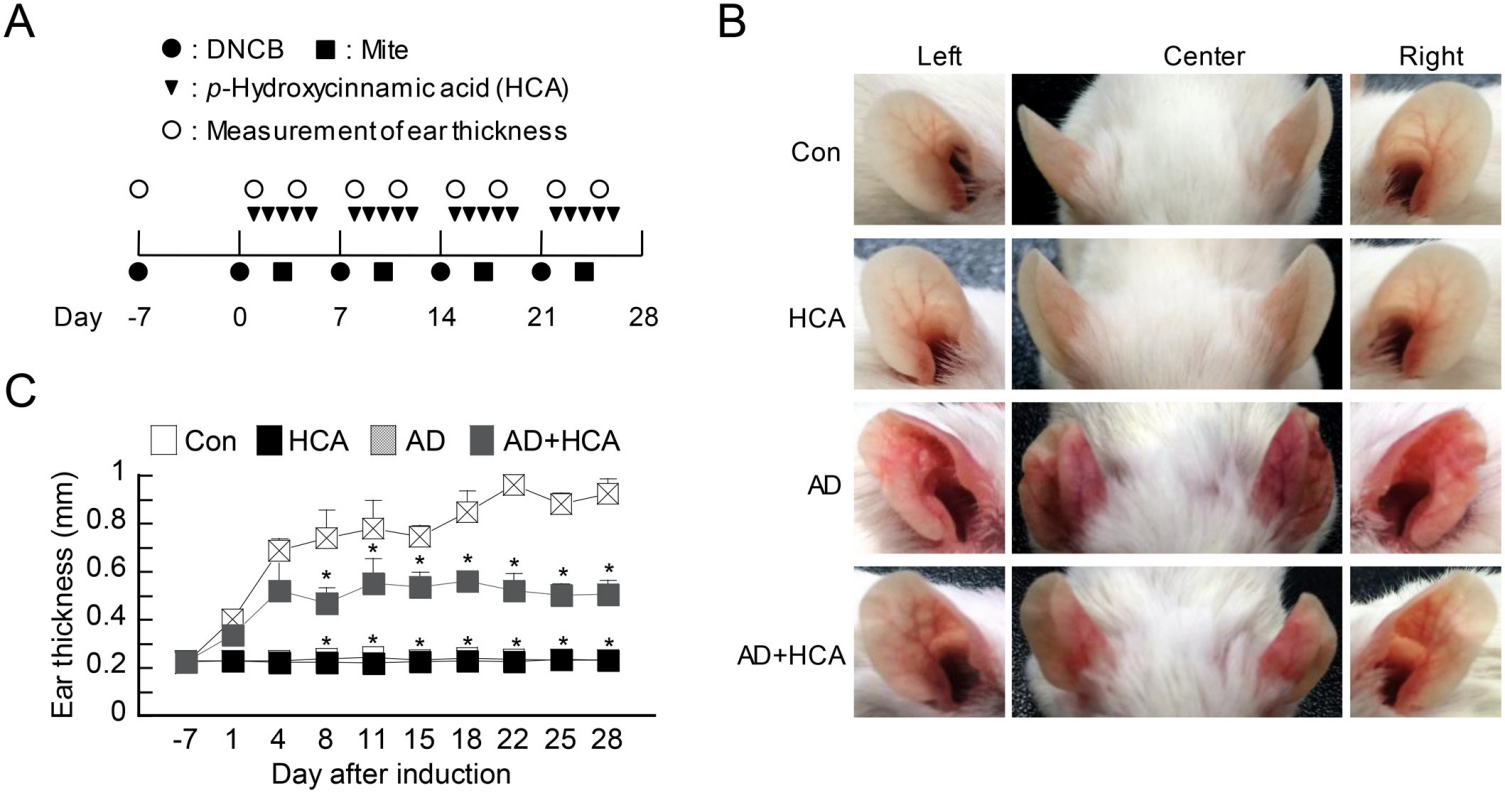
Oral administration of *p*-hydroxycinammic acid attenuates the manifestations of atopic dermatitis in mice. (A) Schematic depiction of the induction and *p*-hydroxycinammic acid (HCA) treatment of atopic dermatitis (AD). There were four mouse groups: healthy untreated controls (Con), mice treated with HCA alone (HCA) or 2, 4-dinitrochlorobenzene/mite extract alone (AD), and mice treated with both (AD+HCA) (n = 3–6 per group). (B) Representative photographs of mouse ears from each group 28 days after starting AD induction (*i*.*e*., 28 days after the first application of 2, 4-dinitrochlorobenzene). (C) Ear thickness during the course of AD. The data are expressed as mean ± SD. *P<0.05, *versus* the AD control group.

AD was induced in BALB/c mice by repeatedly exposing the ears to mite extract and DNCB as described previously [19]. The experimental protocol used in the present study is depicted schematically in Fig 1A. There were four mouse groups, namely, healthy control mice that were not treated with either DNCB/mite extract or HCA, control mice that were treated with DNCB/mite extract alone or HCA alone, and test mice that were treated simultaneously with both DNCB/mite extract and HCA. To induce AD, the surfaces of both ear lobes were stripped five times with a surgical tape (Seo-il chemistry, Hwa-sung, Korea). After stripping, each ear was painted with 20 μl of DNCB (1%). Four days later, the ears were painted with 20 μl of mite extract (10 mg/ml). The mite extract/DNCB treatment was repeated weekly for 4 weeks. HCA treatment consisted of oral HCA administration at a concentration of 50 mg/kg/day. The treatment started 1 day after the second DNCB application and was repeated daily for another 4 days. After a 2 day pause, this 5 days-on and 2 days-off HCA treatment protocol was repeated. This procedure was followed for 4 weeks. Ear thickness was measured 24 h after DNCB or mite extract application by using a dial thickness gauge (Kori Seiki MFG Co., Japan). The animals were sacrificed on day 28. Blood samples were collected by cardiac puncture. Plasma was prepared from the blood samples and stored at -70°C for further analysis. After blood collection, the ears were removed and subjected to histopathological analysis.

### Real-time PCR

Total RNA was isolated from the ear tissues, the CD4^+^ T cells in the spleen, draining lymph nodes (dLNs), and non-dLNs, or the HaCaT keratinocytes by using TRI Reagent (Molecular Research Center, Cincinnati, OH). The RNA was reverse-transcribed by using RT Premix (Enzynomics, Daejeon, Korea) and then subjected to PCR employing the primers listed in Table 1. The amplification protocol consisted of denaturation at 94°C for 30 s, annealing at 60–62°C for 20 s, and extension at 72°C for 40 s. The 30 cycles were preceded by denaturation at 72°C for 7 min. PCR amplification was performed in StepOne Real-Time PCR Systems (Applied Biosystems, Waltham, MA) for continuous fluorescence detection in a total volume of 10 μl of cDNA/control and gene-specific primers by using SYBR Premix Ex Taq (Takara Bio Inc., Shiga, Japan). The mRNA levels of the target genes were normalized relative to GAPDH by using the following formula: relative mRNA expression = 2^- (ΔCt of target gene-ΔCt of GAPDH)^, where Ct is the threshold cycle value.

**Table 1:** PCR primer sequences for mRNA quantification.

mouse *TNFα*	F	5’-aagcctgtagcccacgtcgta-3’
	R	5’-ggcaccactagttggttgtctttg-3’
mouse *IFNγ*	F	5’-tcaagtggcatagatgtggaagaa-3’
	R	5’-tggctctgcaggattttcatg-3’
mouse *IL-4*	F	5’-acaggagaagggacgccat-3’
	R	5’-gaagccgtacagacgagctca-3’
mouse *IL-5*	F	5’-gaagtgtggcgaggagagac-3’
	R	5’-gcacagttttgtggggtttt-3’
mouse *IL-6*	F	5’-ccggagaggagacttcacag-3’
	R	5’-ggaaattggggtaggaagga-3’
mouse *IL-13*	F	5’-gcaacatcaacaggaccaga-3’
	R	5’-gtcagggaatccagggctac-3’
mouse *IL-31*	F	5’-tcggtcatcatagcacatctggag-3’
	R	5’-gcacagtccctttggagttaagtc-3’
mouse *IL-17*	F	5’-tcccctctgtcatctgggaag-3’
	R	5’-ctcgaccctgaaagtgaagg-3’
mouse *TSLP*	F	5’-aggctaccctgaaactgag-3’
	R	5’-ggagattgcatgaaggaatacc-3’
mouse *T-bet*	F	5’-agcaaggacggcgaatgtt-3’
	R	5’-gggtggacatataagcggttc-3’
mouse *GATA3*	F	5’-ctcggccattcgtacatggaa-3’
	R	5’-ggatacctctgcaccgtagc-3’
mouse *GAPDH*	F	5’-gcacagtcaaggccgagaat-3’
	R	5’-gccttctccatggtggtgaa-3’
human *TNFα*	F	5’-cctaccagaccaaggtcaac-3’
	R	5’-agggggtaataaagggattg-3’
human *IL-1β*	F	5’-ggatatggagcaacaagtgg-3’
	R	5’-atgtaccagttggggaactg-3’
human *IL-6*	F	5’-aaagaggcactgccagaaaa-3’
	R	5’-atctgaggtgcccatgctac-3’
human *TSLP*	F	5’-tagcaatcggccacattgcct-3’
	R	5’-gaagcgacgccacaatccttg-3’
human *GAPDH*	F	5’-cggagtcaacggatttggtcgtat-3’
	R	5’-agccttctccatggtggtgaagac-3’

### Histological analysis

The ears of the mouse groups were fixed with 10% paraformaldehyde and embedded in paraffin. The paraffin blocks were sliced into 5 μm-thick sections, deparaffinized, and stained with hematoxylin and eosin (H&E). The H&E-stained slides were used to measure the thickness of the epidermis and dermis. To count the infiltrating mast cells, sliced sections were stained with 0.01% toluidine blue and the mast cells at three randomly chosen sites were counted. To count the infiltrating CD4^+^ T cells in the ear tissues, the paraffinized blocks were sliced and stained with anti-mouse CD4 antibody conjugated with FITC. Fluorescence was measured under a confocal microscope and CD4^+^ T cells were counted at three randomly chosen sites. In nuclear p65 translocation study, the 5 μm-thick serial sections were immunostained with rabbit primary antibodies against mouse keratin or p65, incubated with appropriate secondary antibodies, and then imaged by a confocal microscope. Nuclear p65 translocation in keratinocytes were measured by counting the cells with colocalization of p65 with DAPI at the randomly chosen sites in the KRT5-stained region.

### Measurement of serum Igs

The total IgE, IgG2a, and mite-specific IgE titers in the day 28 sera were measured by using a commercial ELISA kit according to the manufacturer’s instructions.

### T-cell differentiation

Naïve C57BL/6 or BALB/c mice were sacrificed and CD4^+^ T cells were isolated from the LNs and spleens by magnetic-activated cell sorting (MACS) separation (Miltenyi Biotec, Bergisch Gladbach, Germany). The isolated cells were incubated on plates that were pre-coated with anti-mouse CD3 antibody (2 μg/ml) and treated with soluble anti-mouse CD28 antibody (2 μg/ml). To induce Th1 differentiation, the cells were then incubated with a cocktail composed of anti-mouse IL-4 antibody (10 μg/ml), mouse IL-12 recombinant protein (10 ng/ml), and human IL-2 recombinant protein (2 IU). Alternatively, to induce Th2 differentiation, the cells were incubated with a cocktail composed of anti-mouse IFNγ antibody (5 μg/ml), anti-mouse IL-12 antibody (5 μg/ml), mouse IL-4 recombinant protein (10 ng/ml), and human IL-2 recombinant protein (2 IU). In both treatments, hIL-2 was freshly added every day. After 5 days of incubation, the cells were harvested and total RNA was extracted to assess the expression levels of the transcription factors T-bet (Th1) and GATA3 (Th2).

### Intracellular cytokine staining

To determine differentiation efficiency, Th1-polarized and Th2-polarized cells that were generated as described above were cultured in the absence or presence of HCA on 12-well plates (1 × 10^6^). The cells were stimulated with PMA/A23187 for 2 h, after which brefeldin A (10 μg/ml) was added and the cells were incubated for another 2 h. The cells were then harvested, fixed with 4% paraformaldehyde, permeabilized with 0.1% saponin solution, and stained with intracellular cytokine antibodies (anti-mouse IFNγ for Th1-polarized cells and anti-mouse IL-4 for Th2-polarized cells). The samples were washed twice with phosphate-buffered saline and fluorescence was measured by flow cytometry (FACSCanto II, BD Biosciences, NJ, USA). Flowjo software was used for data analysis.

### Proliferation assay

CD4^+^ T cells isolated from the LNs and spleens of naïve C57BL/6 mice were stained with 10 μM CFSE (Molecular Probe, Carlsbad, CA) and left in an incubator at 37°C for 30 min. The CFSE-labeled cells were then cultivated with recombinant cytokines and neutralizing antibodies to induce T cell differentiation as described above in the absence or presence of HCA (50 μM). After 72 h, the Th1-polarized/Th2-polarized cells were collected and the diluted CFSE intensity was analyzed by using a flow cytometer.

### ELISA to measure Th1 and Th2 cytokine production

Differentiated Th1 and Th2 cells (1 × 10^6^ /well) were seeded on a 24-well plate and pre-incubated with HCA (50 μM) for 30 min. Thereafter, the cells were stimulated with anti-CD3/CD28 antibodies or PMA/A23187 for 24 h. The supernatants were collected and the concentrations of IFNγ (from Th1 cells) and IL-4 (from Th2 cells) were measured by using an ELISA duo set kit (R&D, Minneapolis, MN).

### Luciferase assay

HaCaT keratinocytes (2 × 10^6^) were transfected with 100 μl of Amaxa's Nucleofector solution (Amaxa, Cologne, Germany) containing 3 μg of pGL3-NF-κB or pGL3-AP-1 Luc plasmids in combination with pRL-TK, after which the cells were immediately transferred to 2 ml of complete fresh medium and cultured in six-well plates at 37°C. After 24 h of stabilization, the cells were treated with TNFα (10 ng/ml) and IFNγ (10 ng/ml), and incubated for 16 h. The cells were harvested and lysed in a lysis buffer (Promega, Madison, WI). The proteins were extracted and cellular debris was removed by centrifugation at maximum speed for 20 min. Luciferase activity was measured by using a Centro LB 960 Luminometer (Berthold Technologies, Germany) according to the manufacturer's instructions. Relative luciferase activity was presented as fold activity over the highest values (set to 100).

### Statistics

Experiments were performed independently at least three times on different days. The data are presented as mean ± standard deviation. Groups were compared by using unpaired Student’s *t*-tests and one-way analysis of variance. P<0.05 was considered to indicate statistical significance.

## Results

### Oral administration of HCA attenuates AD manifestations in mice

We previously reported that HCA inhibits T-cell activation *in vitro* [16]. To investigate whether HCA has the potential to ameliorate T cell-mediated diseases, we constructed an experimental AD model by using mite extract and 1% DNCB (Fig 1A). There were four mouse groups: healthy control mice, mice treated with HCA alone, mice treated with DNCB/mite extract alone (AD control group), and mice that were treated with oral HCA while AD was being induced. On day 28 when the mice are in the chronic phase of AD, the ear skin of the AD control mice exhibited swelling and redness; by contrast, the HCA-treated AD mice showed only moderate symptoms (Fig 1B). For a more quantitative analysis of AD manifestations during the course of disease, ear thickness was measured twice a week, namely, one day after applying DNCB or mite extract. Compared with the AD control mice, the ear skin of the HCA-treated AD mice was significantly less thick on day 8 and all time points thereafter (Fig 1C). Thus, oral administration of HCA effectively attenuated AD in the skin of the AD model mice.

### Oral administration of HCA reduces tissue inflammation and immune cell infiltration in AD mice

**Fig 2:**
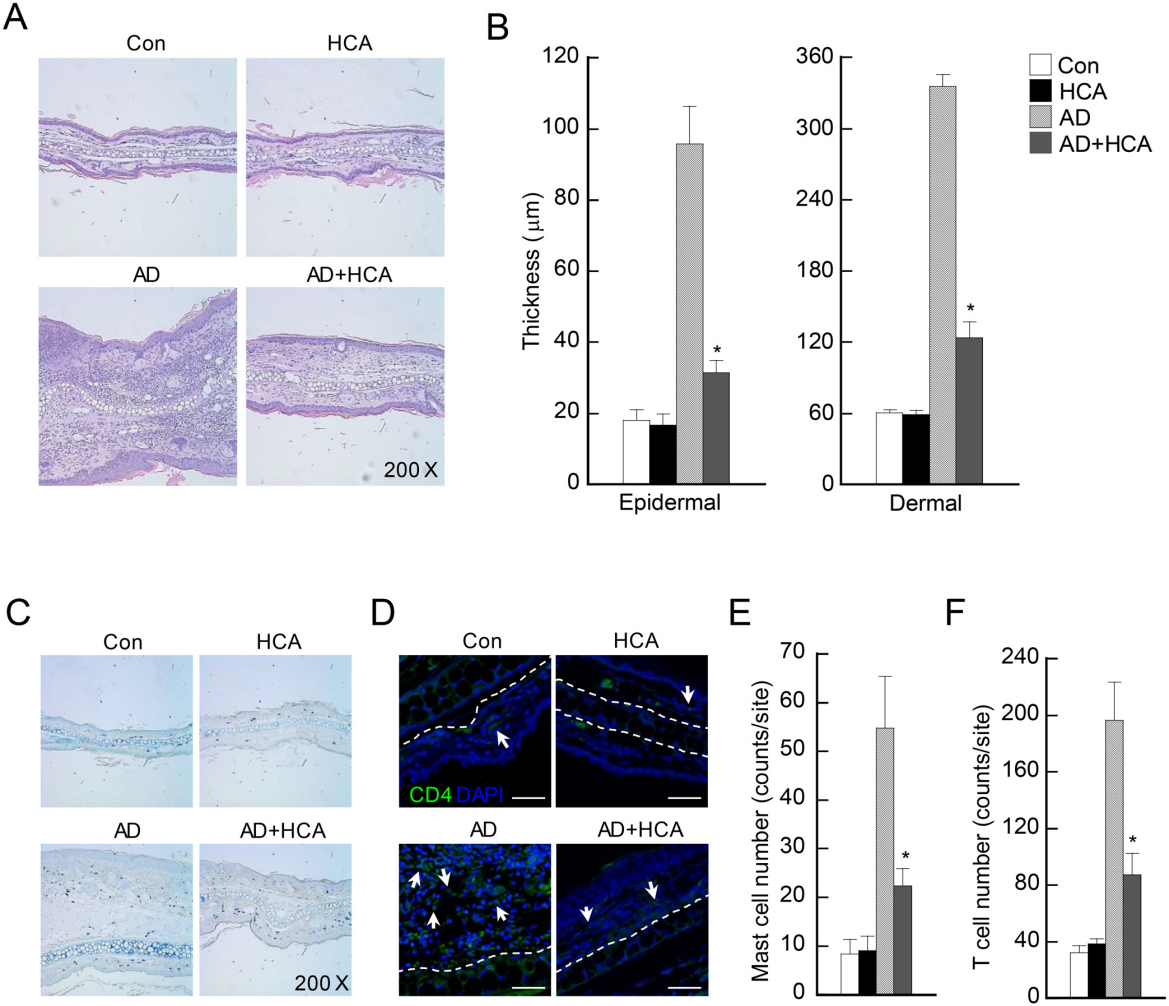
Oral administration of *p*-hydroxycinnamic acid reduces tissue inflammation and immune cell infiltration in atopic dermatitis. (A, C, and D) Microphotographs of sections of the left ear 28 days after the start of atopic dermatitis (AD) induction. The sections were stained with hematoxylin and eosin (H&E) (A), toluidine blue (B), or anti-CD4 antibody (D). Original magnification was X 200. (B) Epidermal and dermal thickness was measured from H&E-stained microphotographs. (E and F) The number of infiltrating mast cells (E) and CD4^+^ T cells (F) in the ear sections, as revealed by toluidine blue and anti-CD4 antibody staining, respectively. At least three randomly chosen sites were analyzed for each cell count experiment. The four mouse groups are described in the legend to Fig 1. The data are presented as the mean ± SD (n = 3–6/group). *P<0.05, *versus* the AD control group.

To determine whether HCA treatment changed the DNCB/mite extract-induced inflammation and immune-cell infiltration in the ear tissues, histological analysis was performed. H&E staining of day 28 ear tissue sections revealed the typical pathological characteristics of AD in the AD mice, namely, hyperkeratosis, acanthosis, and parakeratosis (Fig 2A). The AD control mice showed thickening of the epidermis and dermis; by contrast, the HCA-treated AD mice had significantly less epidermal and dermal ear thickening (Fig 2A and 2B). Toluidine blue staining of the tissue sections indicated the presence of mast cells, while immunofluorescent staining for CD4 molecules indicated the infiltration of T cells. Both mast cells and T cells play critical roles in the development of AD [20,21]. The HCA-treated AD mice had significantly fewer mast and T cells in the ear skin than the AD control mice (Fig 2C–2F). TUNEL assays showed that the AD control and HCA-treated AD mice did not differ in terms of the rates of cell death in the ear, which indicates that the lower mast cell and CD4^+^ T cell counts in the HCA-treated AD mice were not due to HCA-induced cell death (S1A and S1B Fig). Furthermore, when the naïve CD4^+^ T in the thymus were examined by flow cytometry, the HCA control mice were found to have normal development of these cells. By contrast, the AD mice had increased population of CD4^+^ T cells but not CD8^+^ T cells in the thymus, and the naïve CD4^+^ T-cell development in the thymus was altered in the HCA-treated AD mice, indicating that HCA did not impair normal T cell development in the thymus (S2A Fig). Thus, although HCA treatment lowered tissue inflammation and immune-cell infiltration in the experimental AD model, these effects were not due to immune-cell death caused by the long (4 weeks) HCA treatment.

### Oral administration of HCA downregulates the expression of immunoregulatory cytokines in the ear of AD mice

**Fig 3:**
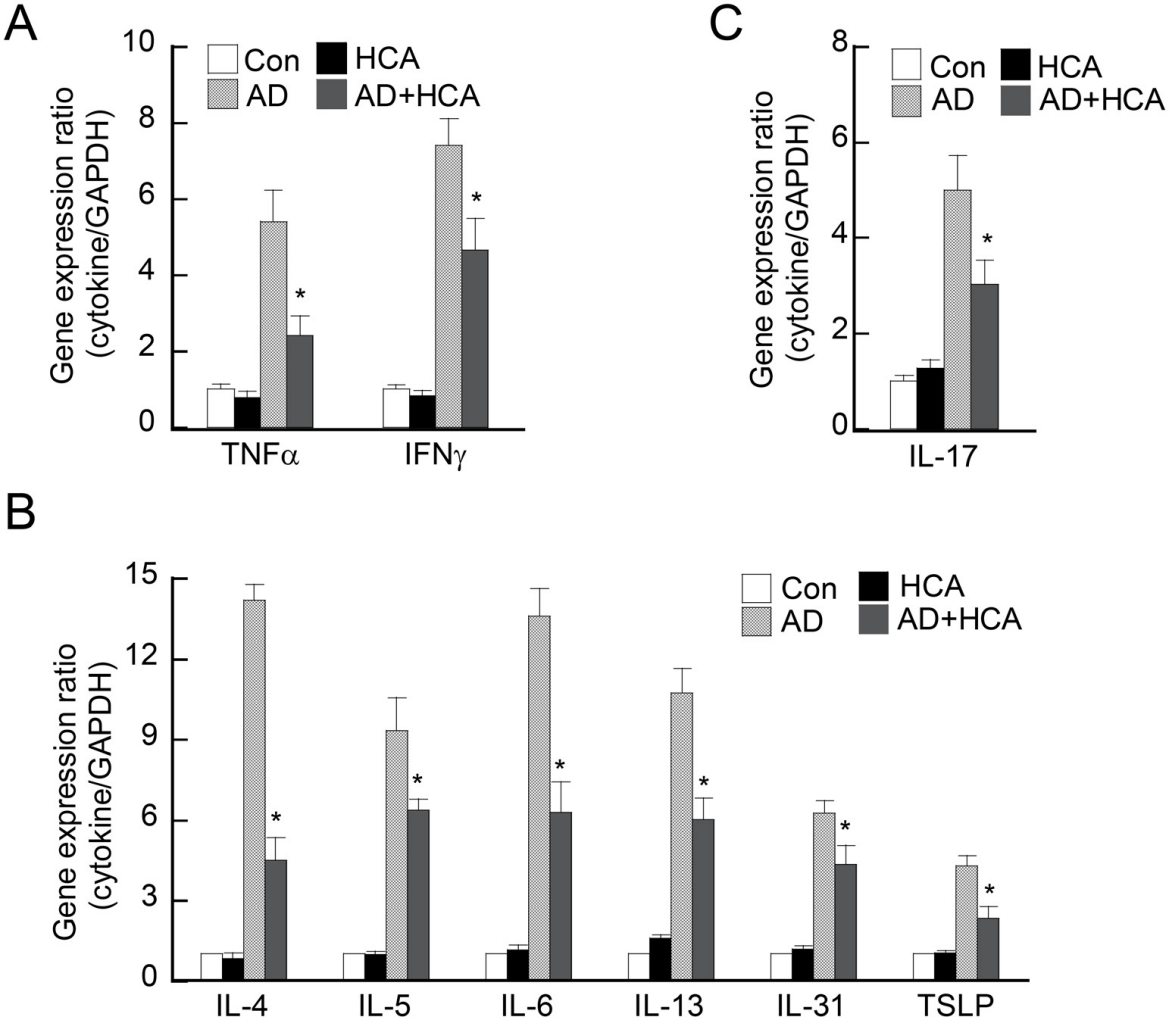
Oral administration of *p*-hydroxycinammic acid downregulates the production of immunoregulatory cytokines in ear tissues in atopic dermatitis. (A–C) The expression of cytokine-encoding genes in the left ear tissues of the mouse groups was analyzed 28 days after atopic dermatitis (AD) was induced. The four mouse groups are described in the legend to Fig 1. Total RNA was isolated from the ear tissues and the gene expression of the indicated cytokines was measured by real-time RT-PCR. The data are presented as mean ± SD (n = 3–6/group). *P<0.05, *versus* the AD control group.

The tissue inflammation in AD mice is secondary to enhanced effector T-cell functions such as the production of pro-inflammatory cytokines. To investigate whether oral administration of HCA suppresses the functions of activated T cells in the ear, the cytokine levels in the day 28 tissues was measured by real-time RT-PCR. As shown by Fig 3A, the HCA-treated AD mice had significantly lower gene expression of the Th1 type cytokines TNFα and IFNγ than the AD control mice. The HCA-treated AD mice also expressed the genes encoding the Th2-type cytokines IL-4, IL-5, IL-6, IL-13, and IL-31 at much lower levels (Fig 3B). The expression of IL-17, which is a typical immunoregulatory cytokine that is released by Th17 cells, was also downregulated in the HCA-treated AD mice (Fig 3C). Similarly, TSLP, which is a keratinocyte-derived cytokine that drives Th2 responses, was significantly downregulated by oral HCA treatment of the AD mice (Fig 3B). Thus, the HCA treatment suppressed the Th2-inducing cytokine production of AD-activated keratinocytes as well. In summary, oral administration of HCA inhibited the production of a large set of immunoregulatory cytokines that are produced by distinct T-cell subsets and keratinocytes.

### Oral administration of HCA attenuates systemic AD manifestations in mice

**Fig 4:**
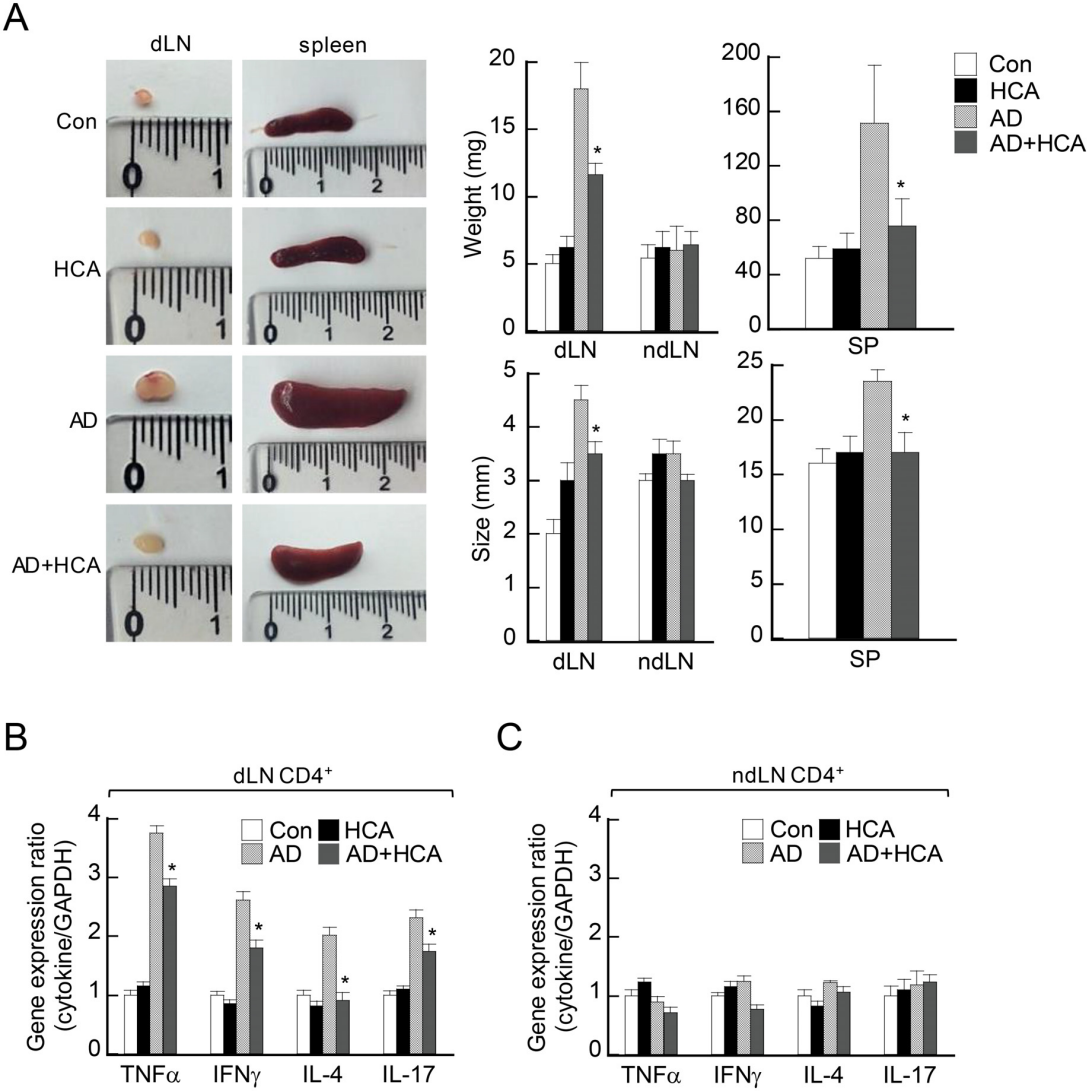
Oral delivery of *p*-hydroxycinammic acid changes the size and weight of immunological organs and downregulates the production of immunoregulatory cytokines by the draining lymph nodes of mice with atopic dermatitis. The four mouse groups are described in the legend to Fig 1. (A) Representative photographs showing the size and weight of the draining lymph nodes (dLN) and spleens of the four mouse groups 28 days after atopic dermatitis (AD) started. The smallest gradation on the ruler is millimeter. The data of the groups (n = 3–6/group) are shown as mean ± SD. *P<0.05 *versus* the AD control group. (B, C). Gene expression of cytokines in CD4^+^ T cells from dLNs (B) and non-dLNs (C) that were harvested on day 28. The CD4^+^ T cells in the lymph nodes were purified by magnetic activated cell sorting according to the manufacturer’s instructions, total RNA was isolated, and the expression of the indicated cytokine-encoding genes was measured by real-time RT-PCR. The data are presented as the mean ± SD (n = 3–6/group). *P<0.05, *versus* the AD control group.

Since AD often develops as a systemic disease [22], we next investigated whether oral administration of HCA influences systemic immune responses. The AD control mice had larger and heavier lymph nodes and spleens than the healthy untreated control mice. By contrast, the oral administration of HCA in AD mice associated with significantly lower dLN and spleen weight and size. Neither AD induction nor HCA treatment, or both, affected the size and weight of the non-dLNs (Fig 4A). Analysis of the gene expression of inflammatory effector cytokines by CD4^+^ T cells purified from dLN revealed that HCA treatment of AD mice associated with significantly lower dLN expression of TNFα, IFNγ, IL-4, and IL-17 (Fig 4B). However, HCA treatment of AD mice had no effect on the cytokine gene expression of the CD4^+^ T cells purified from their non-dLNs (Fig 4C). Thus, oral administration of HCA affected systemic immune responses as well as local responses.

### Oral administration of HCA decreases serum IgE and IgG2a levels in AD mice

**Fig 5:**
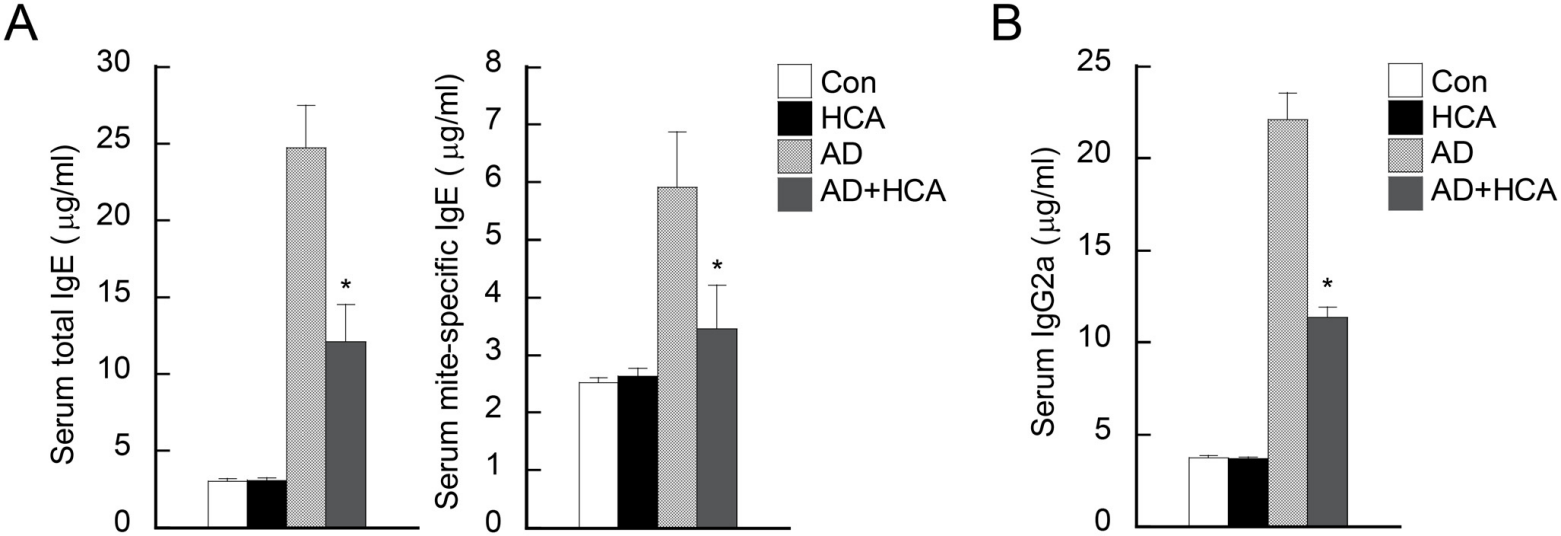
Oral administration of *p*-hydroxycinammic acid decreases the serum IgE and IgG2a levels in mice with atopic dermatitis. The four mouse groups are described in the legend to Fig 1. The total IgE (A, left panel), mite-specific IgE (A, right panel), and total IgG2a (B) levels in the sera from the mouse groups were measured by enzyme-linked immunosorbent assays. Blood samples were collected by cardiac puncture at day 28 post-AD induction. The data are presented as the mean ± SD (n = 3–6/group). *P<0.05, *versus* the AD control group.

Increased serum immunoglobulin (Ig) levels is a hallmark of AD [23]. Indeed, on day 28, the untreated AD mice had high serum levels of total IgE and mite-specific IgE. However, HCA treatment of AD mice associated with significantly lower titers of these Igs (Fig 5A). We also measured serum IgG2a levels because chronic AD often associates with high levels of IgG antibodies and it associates with Th1responses [24]. As expected, the AD control mice had higher IgG2a levels than the healthy normal mice. Oral HCA treatment of AD mice associated with significantly lower IgG2a levels (Fig 5B). The development of B cells in the bone marrow of AD or healthy mice was not affected by HCA treatment (S2B Fig). Thus, HCA inhibited the production of allergen-specific IgE and total IgG2a antibodies.

### HCA inhibits CD4^+^ T-cell differentiation and effector T-cell functions

**Fig 6:**
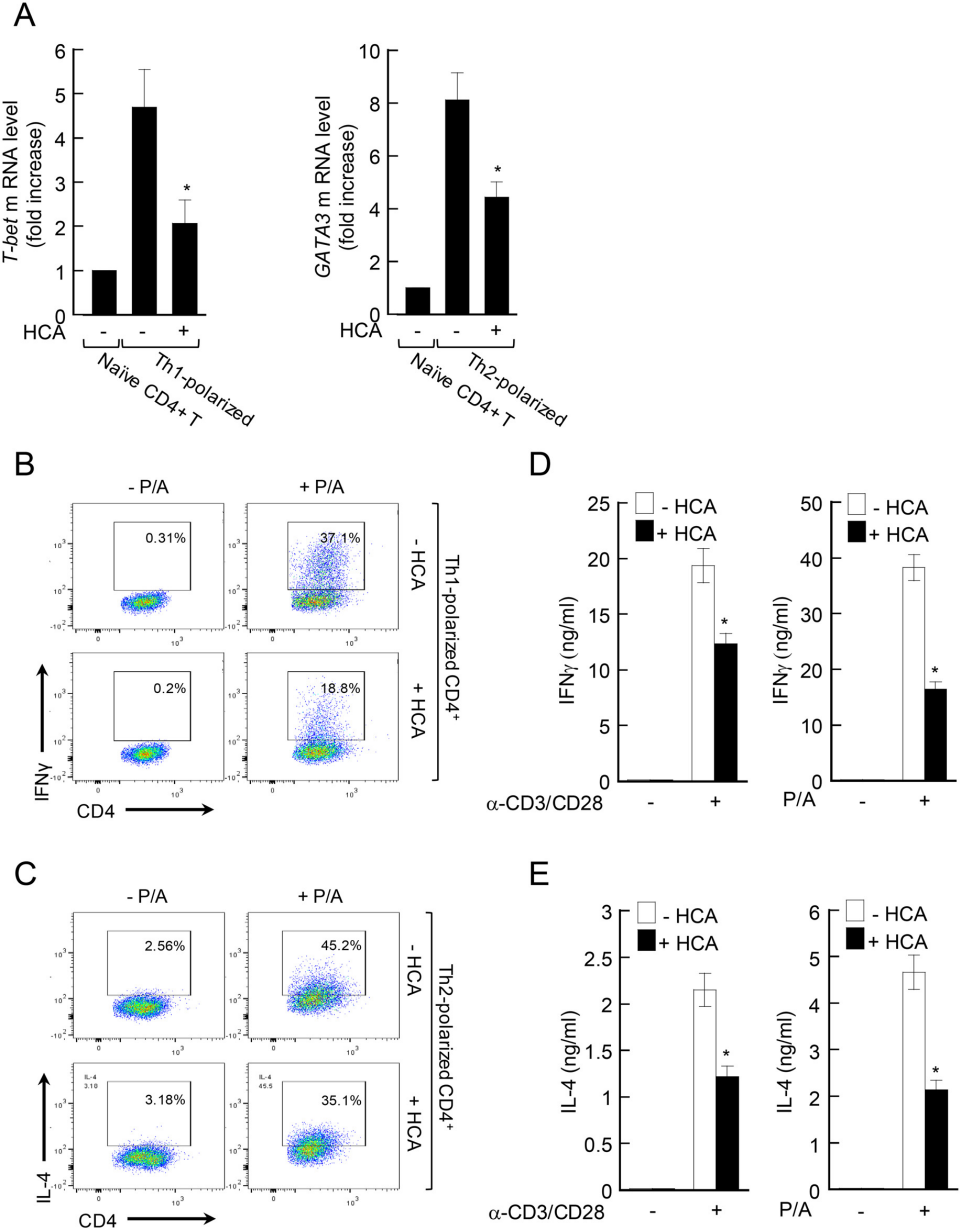
*p*-hydroxycinammic acid inhibits CD4^+^ T cells both before and after differentiation into Th1 and Th2. (A–C) Naïve CD4^+^ T cells from healthy C57BL/6 mice were activated *in vitro* by anti-CD3/anti-CD28 antibodies and induced to differentiate into Th1 or Th2 cells by incubation with the appropriate cocktail of cytokines and anti-cytokine antibodies in the absence or presence of *p*-hydroxycinammic acid (HCA). (A) On day 5 of culture, Th1-polarized cells or Th2-polarized cells were harvested, total RNA was isolated, and the expression of the indicated transcription factors was measured by real-time RT-PCR. (B, C) The Th1-polarized (B) or Th2-polarized (C) cells were treated with PMA/A23187 for 4 h. Two hours before cell harvest, brefeldin A was added. Production of IFNγ (B) or IL-4 (C) was measured by fluorescence-activated cell sorting analysis with intracellular staining. (D, E) Naïve CD4^+^ T cells from normal mice were induced to differentiate as described for A–C. On day 5 of culture, the differentiated Th1 cells (D) or Th2 cells (E) (1 × 10^6^/sample) were pre-incubated with HCA (50 μM) for 30 min and stimulated with anti-CD3/anti-CD28 antibodies (left) or PMA/A23187 (right) for 24 h. The IFNγ (D) and IL-4 (E) levels in the supernatants were measured by using enzyme-linked immunosorbent assays. The data are presented as the mean ± SD (n = 3). *P<0.05, *versus* treatment without HCA.

Proper T-cell differentiation into Th2 cells is pivotal for the development of acute AD [25], whereas switching from Th2 to Th1 in the late phase is critical for the development of chronic AD [6]. Therefore, we next investigated whether HCA regulates the differentiation of T cells from naïve CD4^+^ T cells into Th1 or Th2 *in vitro*. For this, the expression of T-bet and GATA3 was examined because these transcription factors are master regulators of Th1 and Th2 differentiation, respectively. Thus, naïve CD4^+^ T cells were activated by anti-CD3/anti-CD28 antibodies and induced to differentiate into Th1 or Th2 cells by incubating the activated cells with the appropriate cocktail of cytokines and cytokine neutralizing antibodies. When the Th1 or Th2 differentiation occurred in the presence of HCA, the expression of T-bet or GATA3 was downregulated, respectively (Fig 6A). Similar findings were observed in T cells isolated from the LNs and spleens of BL6 or BALB/c mice (data not shown). In addition, HCA treatment of the Th1-polarized and Th2-polarized cells reduced their intracellular levels of their signature cytokine, namely, IFNγ (Fig 6B) and IL-4 (Fig 6C), respectively. Second, we tested whether HCA further regulate effector function of differentiated T cells. HCA pretreatment also reduced the levels of IFNγ and IL-4 in the supernatants of anti-CD3/CD28 antibody- or PMA/A23187-treated Th1 and Th2 cells, respectively (Fig 6D and 6E). Thus, HCA inhibited T-cell differentiation and the effector functions of Th1 and Th2 cells.

### HCA downregulates T-cell proliferation during differentiation into Th1 and Th2 cells

**Fig 7:**
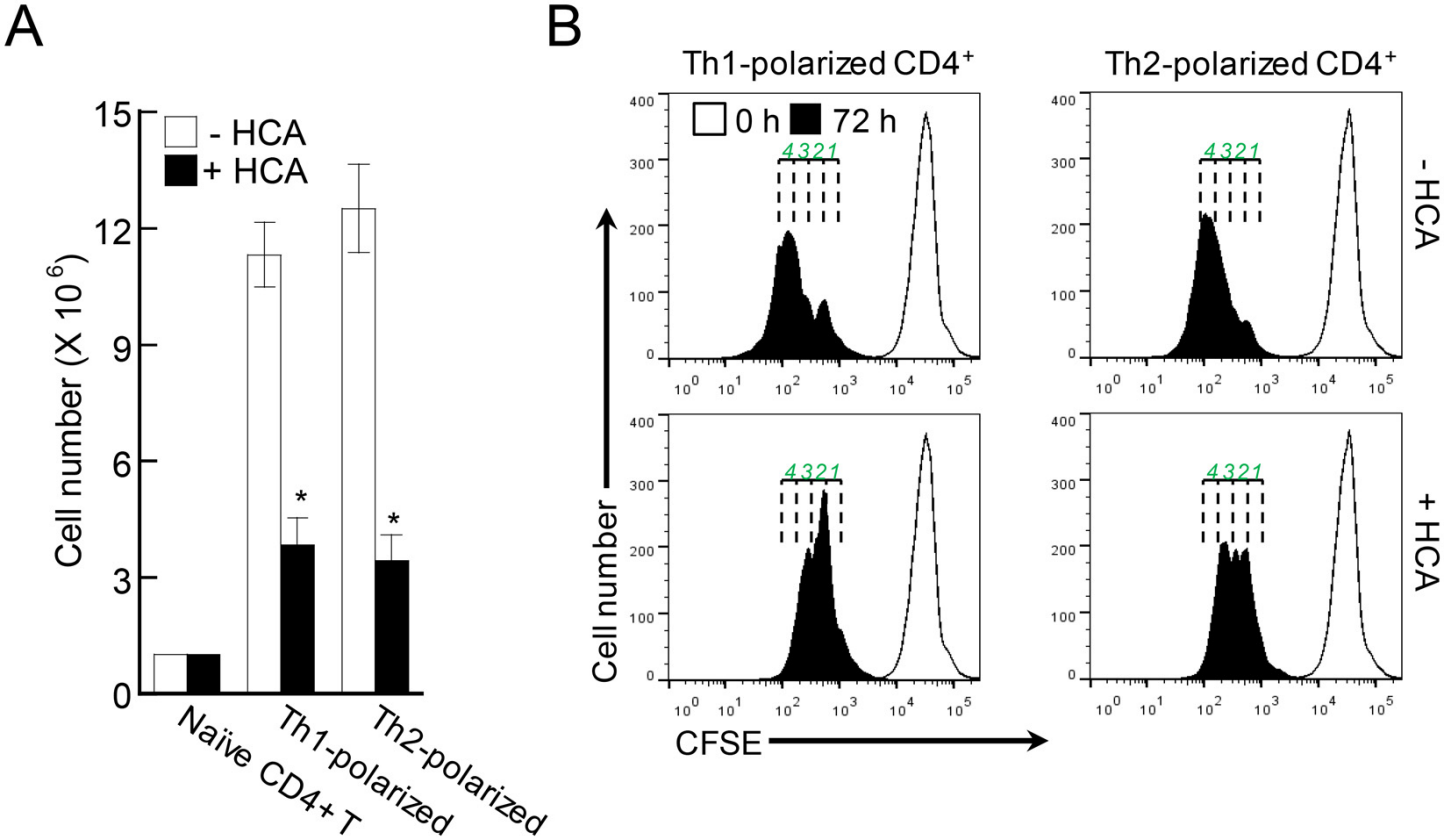
*p*-hydroxycinammic acid downregulates T-cell proliferation during differentiation. (A) Naïve CD4^+^ T cells from LNs and the spleen were induced to differentiate into Th1 and Th2 type effector T cells by adding recombinant cytokines and neutralizing antibodies, in the absence or presence of *p*-hydroxycinammic acid (HCA) (50 μM) for 5 days. On day 5, the cells were harvested and total cell numbers were measured. The data are presented as mean ± SD (n = 3). *P<0.05, *versus* the cells not treated with HCA. (B) CD4^+^ T cells from LNs and spleen were labeled with carboxyfluorescein succinimidyl ester (CFSE, 10 μM) for 30 min and induced to differentiate in the absence or presence of HCA. After 72 h of culture, the cells were harvested and the CFSE intensities were measured by flow cytometry. 0 h indicates naïve CD4^+^ T cells. The numbers above 72 h population indicates the number of cell division. Representative histograms of three independent experiments are shown.

In the present study, we found that HCA treatment *in vitro* significantly reduced the numbers of differentiated Th1 and Th2 cells (Fig 7A). This cannot be due to HCA-induced cell death because our previous work showed that HCA does not induce cell death *in vitro* [16]. Thus, the lower numbers of Th1 and Th2 cells after differentiation in the presence of HCA may reflect HCA-induced inhibition of T-cell proliferation during differentiation. To test this, naïve CD4^+^ T cells were labeled with the fluorescent dye CFSE and then induced to polarize into either Th1 or Th2 cells in the absence or presence of HCA. The fluorescence intensity was measured during the course of polarization. Fig 7B shows that HCA suppressed T-cell proliferation during differentiation. Thus, HCA may decrease the numbers of effector Th1 and Th2 cells by suppressing T-cell proliferation rather than by inducing T-cell death.

### HCA reduces the production of pro-inflammatory cytokines by activated keratinocytes

**Fig 8:**
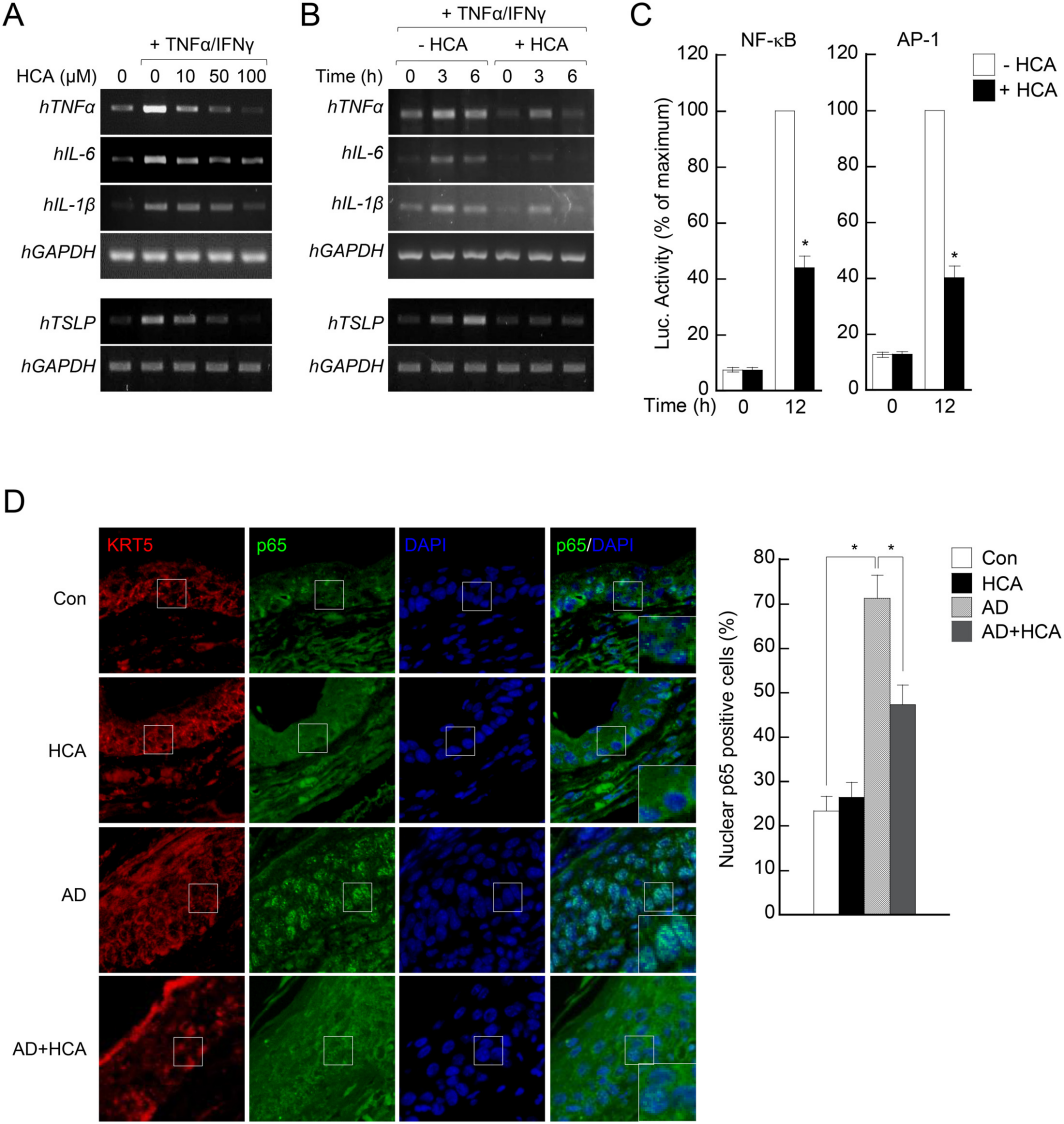
*p*-hydroxycinammic acid reduces the production of proinflammatory cytokines by activated HaCaT keratinocytes by inhibiting NF-κB and AP-1 activity. (A) Human keratinocyte HaCaT cells (1 × 10^6^ /well) were pre-treated with *p*-hydroxycinammic acid (HCA) at different concentrations (10–100 μM) for 30 min and stimulated with tumor necrosis factor (TNF)α (10 ng/ml) and interferon (IFN)γ (10 ng/ml). After 3 h of incubation, the cells were harvested, total RNA was isolated, and the expression of genes encoding inflammatory cytokines (TNFα, IL-1β, IL-6, and TSLP) was measured by conventional RT-PCR. (B) HaCaT cells were pretreated with HCA (50 μM) for 30 min and then activated with TNFα and IFNγ. At different time points (0, 3, and 6 h), the cells were harvested, total RNAs were isolated, and the expression of genes encoding inflammatory cytokines (TNFα, IL-1β, IL-6, and TSLP) was measured by conventional RT-PCR. (C) HaCaT cells (2×10^6^) were transfected with either the pGL3-NF-κB (C) or AP-1 (D) construct in combination with pRL-TK and stabilized for 24 h. The cells were further incubated for 1 h in the absence or presence of HCA (50 μM) and then stimulated for 16 h with TNFα (10 ng/ml) and IFNγ (10 ng/ml). The luciferase activities were measured by a luminometer. The data from three independent treatments are expressed as the mean ±SD. *P<0.05, *versus* mock (A) or *versus* treatment without HCA (B–D). (D) Microphotographs of sections of the left ear 28 days after the start of AD induction. The serial sections were immunostained for either keratin 5 (KRT5) or p65 plus DAPI. Lower inserts (left panels) show the costaining for p65 (green) and DAPI (blue) in the squares. At least three randomly chosen sites (squares) in the KRT5 regions were analyzed for nuclear p65 positive cells on the right panel. The data are expressed as mean ± SD (n = 3/group).

Keratinocytes contribute to the development of AD by producing multiple proinflammatory cytokines [21,22,23]. Since oral HCA treatment attenuated the morphological features of ear keratinocytes in the AD mice (Fig 2A), we next tested whether HCA treatment directly affects keratinocytes *in vitro and in vivo*. Thus, HaCaT keratinocytes were pretreated with an increasing dose of HCA (0–100 μM) for 30 min before being stimulated with IFNγ and TNFα for 16 h. As shown in Fig 8A, the cytokine-activated keratinocytes produced high levels of the proinflammatory cytokines TNFα, IL-6, IL-1β, and TSLP. However, HCA pretreatment suppressed this production of proinflammatory cytokines by the keratinocytes in an HCA dose-dependent manner. Moreover, time-course experiments showed that HCA strongly inhibited the keratinocyte production of cytokines early after the cells were stimulated (*i*.*e*., after ~3 h of stimulation) with TNFα and IFNγ (Fig 8B). Similarly, the HCA treatment of keratinocytes dramatically suppressed the activities of NF-κB and AP-1, which are key transcription factors that govern the transcription of proinflammatory mediators (Fig 8C). Consistent with the *in vitro* findings, we found that oral administration of HCA directly affects the keratinocytes in AD model. The AD mice showed a thick layer of keratinocytes with keratin 5 (KRT5), a cytoplasmic intermediate filament protein produced by skin epithelial cells. In keratinocytes of AD mice displayed a marked increase in p65 nuclear translocation, which was significantly blunted in HCA-treated AD mice, indicating that oral administration directly influences keratinocytes in AD mice (Fig 8D). These results suggest that HCA may be able to exert global control over an entire set of immunoregulatory genes in keratinocytes. All together, these observations show that HCA regulates both T-cells and keratinocytes in AD.

## Discussion

This present study examined the anti-AD potential of an orally administered phenolic compound from *C*. *longa* by using an animal model of AD. Oral administration of effective compounds is of particular interest in the AD field because topical administration delivers the compound to the lesions rapidly and directly, it can be difficult to control the dosage. This can either reduce the efficacy of the compound or lead to adverse side effects. By contrast, oral administration allows close control of the dose over long-term periods. This is particularly important for adequately treating children or non-compliant patients in the clinic. Moreover, AD is a chronic disease that is manifested not only by local symptoms but also by profound systemic immune system changes that drive disease. Therefore, the best approach to treating AD would be to administer anti-AD compounds orally at a dose that is both effective and does not induce side-effects. The present study showed that when mice were treated orally with HCA, both the local and systemic AD manifestations were ameliorated without inducing any significant effects on the normal immune responses. Specifically, 4 weeks of HCA treatment attenuated the pathological features in the skin, dLNs, spleen, and blood of mice with AD without altering the basal levels of IgE in the serum of healthy mice and the basal cytokine production by T-cells in the dLNs.

Our main observation was that oral administration of HCA alleviated morphological manifestations in the skin of AD mice: while the DNCB/mite extract induced epidermal and dermal thickening (which may be due to dysregulation of keratinocyte functions [26]), HCA treatment associated with much less skin thickening. This suggested that HCA could prevent allergen-induced derangement of keratinocyte functions. Indeed, a closer analysis revealed that HCA treatment reduced the production of proinflammatory cytokines (TSLP, TNFα, IL-6 and IL-1β) by keratinocytes in both the ear tissues and *in vitro*. This is significant because keratinocytes play a pivotal role in the development of AD. First, they produce the chemokine CCL17 in the AD skin lesion, which promotes the migration to the lesion of Th2 cells that express CCR4 [27]. Second, they also produce TSLP, which regulates the migration, activation and maturation of multiple key players, including T cells and dendritic cells, in AD [28]. Third, keratinocytes secrete the classical proinflammatory cytokines. Notably, the production of those cytokines is regulated by the transcriptional factors NF-κB and AP-1 [29]. Both NF-κB and AP-1 are ubiquitously expressed and play critical roles in the response of cells to cellular stress (i.e. inflammatory stimuli) that can induce cell activation and cytokine production. We found both previously and in the present study that HCA treatment inhibited the transcriptional activities of these two factors in both keratinocytes (Fig 8C and 8D) and in T cells [16]. This property of HCA is responsible for the suppressive effects of HCA on the proinflammatory cytokine production by keratinocytes and T cells that lead to skin lesion amelioration in AD.

Another mechanism by which HCA may exert its suppressive effects on keratinocytes and T cells in AD involves the cytosolic protein PKC. HCA regulates cytosolic proteins directly [16] and PKC is one of the candidate HCA-targeted molecules. We showed previously that HCA inhibits the phosphorylation of PKC-θ [16] and the activity of PKC in T cells [9]. Moreover, structural approaches indicate that HCA may bind to the active sites of multiple PKC isoforms, thereby blocking PKC activity. PKC activation involves a diverse range of signaling pathways that lead to cell activation, proliferation, differentiation, migration, and gene expression. In particular, PKC-θ in T cells is pivotal in TCR-mediated activation [30] and is known to play a key role in regulating the development of Th2-type diseases such as AD [31]. Notably, PKC-α is also expressed by keratinocytes and functions as a critical regulator of keratinocyte activation and production of cytokines [32]. It is possible that HCA inhibits PKC activity in keratinocytes *via* the same or a similar mechanism by which HCA modulates PKC activity in T cells [16] (namely, inhibition of the activation of PKC isoforms). By doing so, HCA can regulate the interplay of T cells, mast cells, dendritic cells, and keratinocytes that drives AD, thus alleviating the local and systemic manifestations of AD.

Further studies that elucidate whether HCA regulates both T cells and keratinocytes by inhibiting PKC isoforms and/or by suppressing NF-κB and AP-1 activity are warranted. These studies are likely to promote the development of effective therapeutics for a wide range of skin inflammatory disorders.

## Supporting Information

S1 Fig HCA does not induce cell death of immune cells in the AD mice. (PDF)

S2 Fig HCA does not impair T and B cell development in the primary lymphoid organs. (PDF)
