# Supplementary material for: Oral Administration of p-Hydroxycinnamic Acid Attenuates Atopic Dermatitis by Downregulating Th1 and Th2 Cytokine Production and Keratinocyte Activation
Source: PLoS One. 2016 Mar 9;11(3):e0150952. doi: 10.1371/journal.pone.0150952 (PMC4784746; doi:10.1371/journal.pone.0150952)
Supplement: S1 Fig — (PDF) [file pone.0150952.s001.pdf]

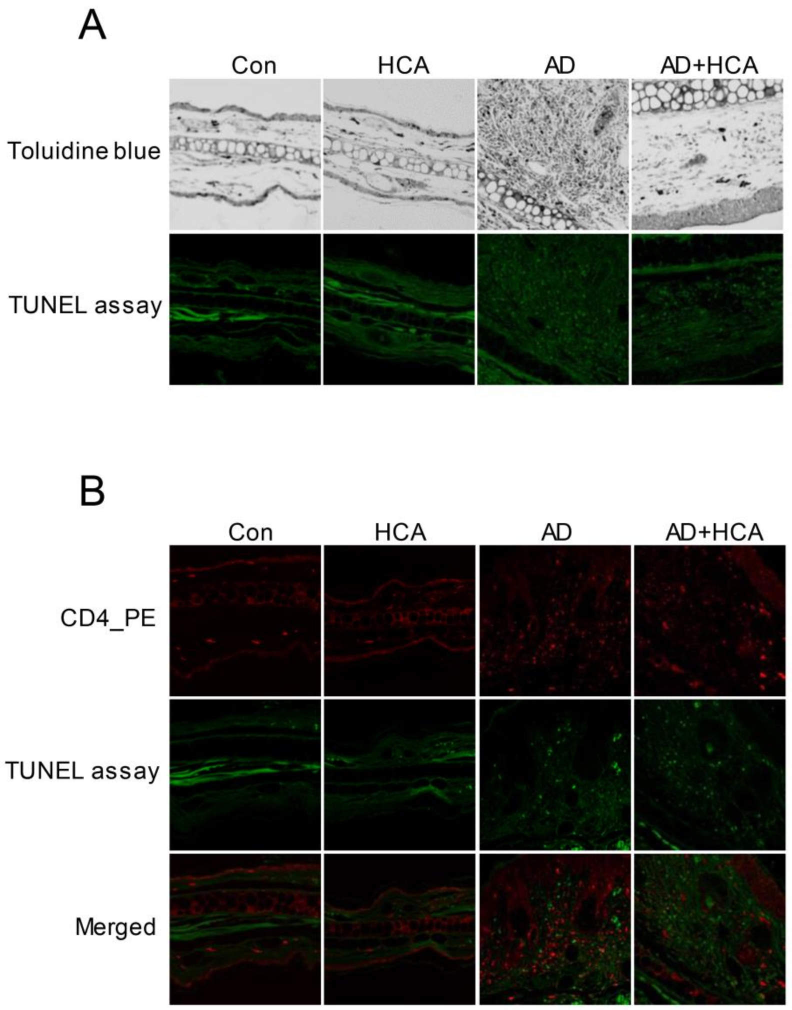

S1 Figure. HCA does not induce cell death of immune cells in the AD mice.

(A and B) The sections of ear tissues from normal or AD mice receiving HCA or left untreated were subjected to either toluidine blue stain or PE-anti-CD4 antibody staining, in combination with TUNEL apoptosis detection assays to assess apoptosis of mast cells or CD4 T cells between before and after HCA treatment. Representative images are shown (n=3/group).
