# Supplementary material for: Oral Administration of p-Hydroxycinnamic Acid Attenuates Atopic Dermatitis by Downregulating Th1 and Th2 Cytokine Production and Keratinocyte Activation
Source: PLoS One. 2016 Mar 9;11(3):e0150952. doi: 10.1371/journal.pone.0150952 (PMC4784746; doi:10.1371/journal.pone.0150952)
Supplement: S2 Fig — (PDF) [file pone.0150952.s002.pdf]

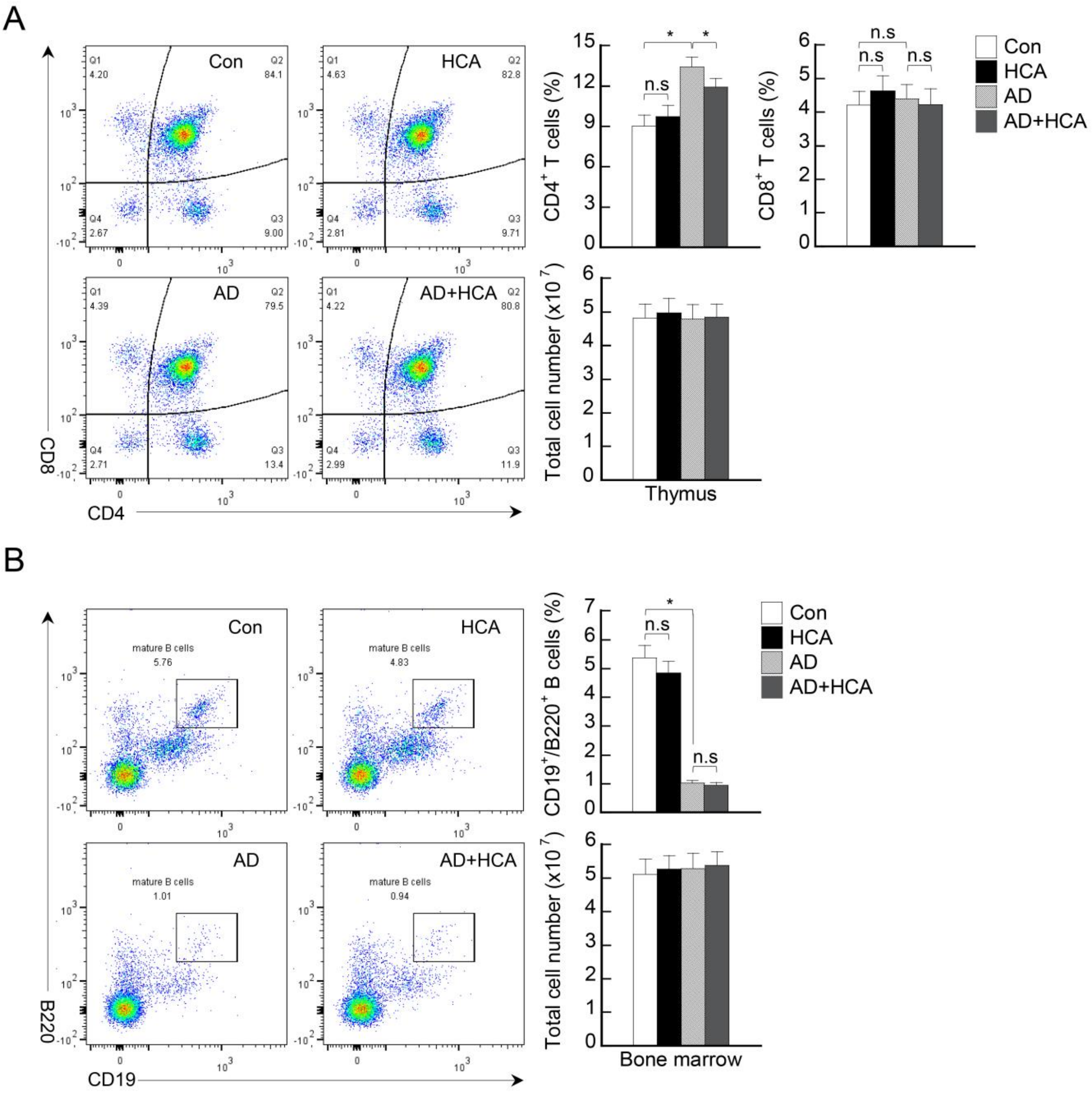

S2 Figure. HCA does not impair T and B cell development in the primary lymphoid organs. (A and B) During AD, mice were administered with HCA for 4 weeks. Thymus and bone marrow were isolated and the cells were counted and subjected to staining for CD4 and CD8 (in the cells from thymus, A) or for B220 and CD19 (in the cells from bone marrow, B). Representative plots are shown. Data are presented as the mean ± SD from three independent experiments.
